# Structural grey matter changes in the substantia innominata in Alzheimer's disease and dementia with Lewy bodies: a DARTEL‐VBM study

**DOI:** 10.1002/gps.4500

**Published:** 2016-05-19

**Authors:** Sean J. Colloby, Greg J. Elder, Riham Rabee, John T. O'Brien, John‐Paul Taylor

**Affiliations:** ^1^ Institute of Neuroscience, Newcastle UniversityCampus for Ageing and VitalityNewcastle upon TyneUK; ^2^ Department of PsychiatryUniversity of CambridgeCambridgeUK

**Keywords:** DARTEL‐VBM, dementia with Lewy bodies, Alzheimer's disease, magnetic resonance imaging, substantia innominata

## Abstract

**Objectives:**

Several cholinergic nuclei, and in particular the nucleus basalis of Meynert, are localised to the substantia innominata in the basal forebrain. These nuclei provide major cholinergic innervation to the cerebral cortex and hippocampus, and have an essential role in cognitive function. The aim of this study was to investigate volumetric grey matter (GM) changes in the substantia innominata from structural T1 images in Alzheimer's disease (AD), dementia with Lewy bodies (DLB) and healthy older participants using voxel‐based morphometry.

**Methods:**

Participants (41 DLB, 47 AD and 39 controls) underwent 3 T T1 magnetic resonance imaging and cognitive assessments. Voxel‐based morphometry analysis used SPM8 with a substantia innominata brain mask to define the subspace for voxel GM analyses. Group differences, and selected behavioural and clinical correlates, were assessed.

**Results:**

Compared with that in controls, bilateral GM loss in the substantia innominata was apparent in both AD and DLB. Relative to controls, significant bilateral GM loss in the substantia innominata was observed in DLB and AD. In DLB, significant associations were also observed between substantia innominata GM volume loss, and the levels of cognitive impairment and severity of cognitive fluctuations.

**Conclusions:**

Relative to that controls, atrophy of the substantia innominata was apparent in DLB and AD, and is associated with specific clinical manifestations in DLB. © 2016 The Authors. *International Journal of Geriatric Psychiatry* Published by John Wiley & Sons Ltd.

## Introduction

Dementia with Lewy bodies (DLB) is a common type of dementia, which accounts for up to 20% of cases at post‐mortem (Holmes *et al*., 1999). Patients with DLB display a range of symptoms including cognitive and visuospatial deficits and, in particular, exhibit the characteristic symptom of fluctuating cognition (McKeith, 2006; McKeith *et al*., 2005). These features are associated with the occurrence of other commonly observed neuropsychiatric DLB symptoms, including visual hallucinations (Collerton *et al*., 2005). Whilst there is a significant overlap between DLB and Alzheimer's disease (AD) in terms of symptoms, in the early stages of DLB, individuals typically display more severe visuoperceptual, attentional and executive function deficits, with relative preservation of memory, compared with similarly impaired AD patients (Metzler‐Baddeley, 2007). The manner in which the cholinergic pathways are affected in DLB compared with other dementias may explain these differences. For example, when compared with those with AD and Parkinson's disease (PD), individuals with DLB show a more marked loss of cholinergic neurons and greater alterations in cortical and subcortical cholinergic receptors (Francis and Perry, 2007; Tiraboschi *et al*., 2002). The cholinergic deficits shown in DLB may also influence prominent symptoms such as visual hallucinations (Onofrj *et al*., 2013; Perry *et al*., 1990b; Tiraboschi *et al*., 2002), and the importance of these deficits upon cognitive symptoms is reinforced by the greater response to cholinesterase inhibitors in this group compared with AD (Aarsland *et al*., 2004).

Cholinergic deficits in DLB are likely to be driven by the degeneration of cholinergic neurons within the basal forebrain (Lippa *et al*., 1999). The substantia innominata (SI) forms part of the basal forebrain and predominantly contains the nucleus basalis of Meynert (NBM), where the cholinergic nuclei in the NBM are the main source of cholinergic innervation and project to cortical areas (Gratwicke *et al*., 2013, 2015). The NBM has an essential role in cognitive and attentional function (Baxter and Chiba, 1999; Niewiadomska *et al*., 2009) and is therefore likely to have an important patho‐aetiological role in DLB. However, the cholinergic basis of cognitive and attentional dysfunction, and the relationship of the NBM with these features, has not been well‐researched in DLB, with only a limited number of studies to date. For example, Grothe *et al*. (2014) observed that NBM, but not rostral basal forebrain volume, was reduced in DLB compared with AD, and that the NBM volume was also associated with visuoperceptual function in the former. Previous studies have assessed the SI in DLB and AD using magnetic resonance imaging (MRI), where grey matter (GM) loss was observed relative to controls, and was more apparent in AD compared with DLB (Whitwell *et al*., 2007). In contrast, Hanyu *et al*. (2007) observed SI thinning in AD and DLB compared with controls, where this was more marked in AD patients.

It is unclear to what extent cognitive deficits and cognitive fluctuations depend upon the NBM in DLB, despite evidence to suggest the involvement of cholinergic dysfunction (McKeith *et al*., 2000; Pimlott *et al*., 2006). Therefore, the aims of the present study were to perform a GM evaluation of the SI in AD, DLB and healthy older individuals, and to examine their clinical correlates using diffeomorphic anatomical registration through exponentiated lie algebra voxel‐based morphometry (DARTEL‐VBM).

## Methods

### Participants

A total of 127 participants (*M*_age_ = 78.26 years; *SD*_age_ = 7.33 years), including 47 with probable AD (McKhann *et al*., 1984), 41 with probable DLB (McKeith *et al*., 2005) and 39 similarly‐aged healthy controls, were recruited from a community‐dwelling population of patients referred to local Old Age Psychiatry, Geriatric Medicine or Neurology Services. Control participants were recruited from friends and spouses of patients and from a bank of volunteer participants held by the university and local clinical research network. Participants were recruited from two separate studies: Study 1 (31 AD, 23 DLB and 23 controls) and Study 2 (16 AD, 18 DLB and 16 controls).

Exclusion criteria for all participants included contraindications for MRI, a previous history of alcohol or substance misuse, significant neurological history or psychiatric illness, focal brain lesions or the presence of other severe or uncontrolled medical illness, which was verified through the examination of participant medical records. All participants, or where appropriate, their nearest relative, provided written informed consent, and the study was approved by the local research ethics committee.

### Measures

Assessment of global cognitive measures included the mini‐mental state examination (MMSE; Folstein *et al*., 1975) and the Cambridge Cognitive Examination (CAMCOG; Roth *et al*., 1986). Motor parkinsonism was measured with Part III of the Unified Parkinson's Disease Rating Scale (UPDRS‐III; Goetz *et al*., 2008). For individuals with dementia, neuropsychiatric features were assessed using the Neuropsychiatric Inventory (NPI; Cummings *et al*., 1994), where symptom frequency and severity across a range of domains (e.g. depression, anxiety and hallucinations) were rated by a carer/informant, providing a total score as a marker of symptom severity. In the current study, the NPI total score and hallucinations sub‐score were specifically examined (McKeith *et al*., 2000). Cognitive fluctuations were assessed using the Clinician Assessment of Fluctuations (CAF) Scale (Walker *et al*., 2000).

### Magnetic resonance imaging

All participants underwent clinical and neuropsychological assessments, before undergoing T1‐weighted magnetic resonance (MR) scanning on a 3 T MRI system using an eight‐channel head coil (Intera Achieva scanner, Philips Medical Systems, Eindhoven, the Netherlands). Participants were scanned using one of two similar T1 sequences: Study 1, whole brain, three‐dimensional (3D) magnetisation‐prepared rapid acquisition gradient echo (MPRAGE), sagittal acquisition, matrix size 216 (anterior–posterior) × 208 (superior–inferior) × 180 (right–left), repetition time = 8.3 ms, echo time = 4.6 ms, inversion time = 1250 ms, flip angle = 8°, SENSE factor = 2, voxel output 1 × 1 × 1 mm^3^; Study 2, whole brain, 3D MPRAGE, sagittal acquisition, matrix size 240 (anterior–posterior) × 240 (superior–inferior) × 150 (right–left), repetition time = 9.6 ms, echo time = 4.6 ms, inversion time = 1250 ms, flip angle = 8°, SENSE factor = 2, voxel output 0.94 × 0.94 × 1.2 mm^3^.

### Image analysis

DARTEL‐VBM analysis was conducted using SPM8 (http://www.fil.ion.ucl.ac.uk/spm), running on matlab 7.9 (MathWorks, Natick, MA, USA). First, MR images were segmented into GM, white matter (WM) and cerebrospinal fluid (CSF) using SPM8's standard unified segmentation module (Ashburner and Friston, 2005). Second, a GM population template was derived from the entire image dataset using the DARTEL technique (Ashburner, 2007). Third, after an initial affine registration of the DARTEL template to the GM tissue probability map in Montreal Neurological Institute (MNI) space (http://www.mni.mcgill.ca/), nonlinear warping of the segmented images was then performed to match the MNI space DARTEL template. Fourth, GM images were then Gaussian smoothed (8 mm full width at half maximum) and modulated, re‐establishing the original tissue volume prior to spatial normalisation. The voxel size of processed images was 1.5 × 1.5 × 1.5 mm^3^.

### Determination of the substantia innominata brain mask

**Figure 1:**
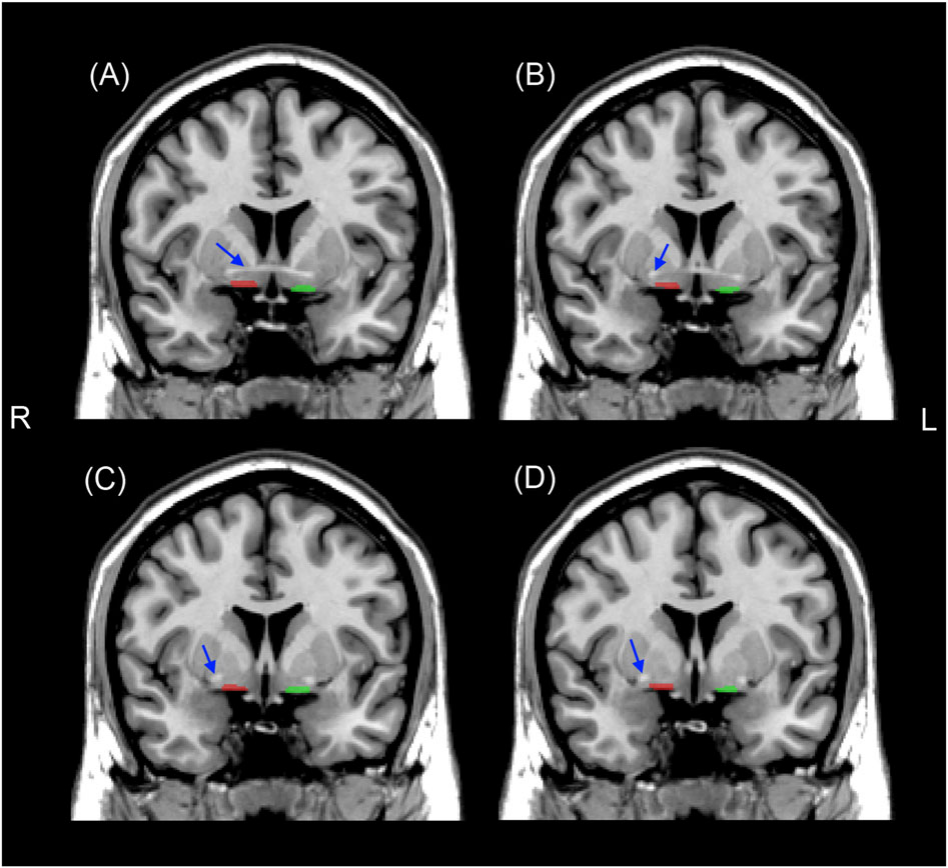
Generation of left (green) and right (red) substantia innominata brain masks on a magnetic resonance imaging T1 brain template for voxel grey matter analyses. Masks were derived from four contiguous coronal sections (anterior → posterior) starting at the level of the hemispheric crossing of the anterior commissure (A), ending at the level at which the anterior commissure fully emerges from the temporal lobe (D). Red and green regions show the segmentations containing the substantia innominata. Blue arrows depict the location of the anterior commissure. R, right; L, left. [Colour figure can be viewed at wileyonlinelibrary.com]

In order to perform a voxel GM assessment of the SI, an MNI space brain mask was derived by a single operator (S. J. C.) manually delineating the boundaries of this structure on coronal sections of a T1‐weighted MRI brain template image (Figure 1(A–D)). The procedure was based on a previously reported protocol demonstrating relatively high intrarater (0.87–0.95) and interrater (0.81–0.89) reliability (Choi *et al*., 2012; George *et al*., 2011; Shin *et al*., 2012). In brief, starting at the coronal slice where hemispheric crossing of the anterior commissure was visible (Figure 1(A)), the ventral globus pallidus and base of the brain including the anterior perforated space set the dorsal and ventral borders of the SI, respectively. The medial border of the SI was defined by a vertical line extending downwards from the ventrolateral aspect of the stria terminalis. The lateral border extended to the medial aspect of the putamen. Definition of the SI borders was then applied to all four contiguous brain slices (anterior → posterior), ending at the level the anterior commissure fully emerges from the temporal lobe (Figure 1(D)). Sizes of the left and right SI segmentations were 111 and 122 voxels, respectively (template voxel size 1 × 1 × 1 mm^3^).

### Statistical analysis

The CAMCOG, MMSE, CAF and UPDRS‐III scores were compared between AD, DLB and controls using one‐way analysis of variance. Group differences in gender, and the proportion of individuals taking cholinesterase inhibitor medications, were compared using chi‐squared tests. Group differences in GM volume were assessed using the general linear model in SPM8, and statistical significance was estimated from the distributional approximations of Gaussian random fields (Friston *et al*., 1994). Age and total intracranial volume (GM + WM + CSF and TIV_SPM8_) were entered into the design matrix as nuisance variables. Multiple regression analyses were also performed to investigate effects of GM loss on clinical and cognitive variables separately in AD and DLB. Predictors entered into the regression model included age, TIV_SPM8_ and the variable of interest. The SI binary mask image defined the brain volume subspace for voxel analyses. Statistical maps were set at a threshold of *p*_uncorrected_ ≤ 0.05 and interpreted as significant if their voxel family‐wise error (FWE)‐corrected *p*‐value within the SI volume subspace (*p*_FWE_) was ≤0.05.

## Results

### Participant characteristics

As expected, CAMCOG and MMSE scores were similar between AD and DLB but significantly differed from controls (Table 1). UPDRS‐III scores were significantly higher in DLB compared to AD and controls. Total NPI, NPI hallucinations and CAF scores were all significantly higher in DLB than AD. The proportion of individuals receiving cholinesterase inhibitors did not significantly differ between dementia groups.

**Table 1:** Demographic and group characteristics

	Control (*n* = 39)		AD (*n* = 47)		DLB (*n* = 41)			
	Mean	SD	Mean	SD	Mean	SD	Test statistic	*p*‐value
Gender (male/female)	25/14		33/14		26/15		*χ* ^2^ = 0.6	0.8
ChEI use (yes/no)	N/A		40/7		32/9		*χ* ^2^ = 0.7	0.4
Age (years)	77.0	6.4	79.0	8.8	78.6	6.2	*F*_(2, 124)_ = 0.8	0.4
MMSE	29.0	1.0	20.8	4.0	20.9	5.0	*F*_(2, 124)_ = 62.0	<0.001^a^
CAMCOG	96.5	3.3	67.8	13.5	69.5	14.9	*F*_(2, 124)_ = 74.1	<0.001^a^
UPDRS‐III	1.2	1.7	2.6	2.4	24.4	13.7	*H*_2_ = 86.1	<0.001^b^
NPI (total)	N/A		9.3	8.7	13.5	11.0	*U*_88_ = 1026.5	0.04
NPI (hallucinations)	N/A		0.2	0.8	2.1	2.1	*U*_88_ = 1521.0	<0.001
CAF	N/A		1.5	3.0	5.9	4.7	*U*_88_ = 1380.5	<0.001
TIV_spm8_ (mL)	1500.0	133.8	1495.4	134.0	1525.0	154.4	*F*_(2, 124)_ = 0.4	0.6

SD, standard deviation; ChEI, cholinesterase inhibitor; MMSE, mini‐mental state examination; CAMCOG, Cambridge Cognitive Examination; NPI, Neuropsychiatric Inventory; UPDRS‐III, Unified Parkinson's Disease Rating Scale (Section III); TIV, total intracranial volume; CAF, Clinical Assessment of Fluctuations; AD, Alzheimer's disease; DLB, dementia with Lewy bodies; N/A, not applicable.

a *Post hoc* test: Controls > AD, DLB (*p* < 0.001), AD versus DLB (*p* > 0.90) (Gabriel's).

b *Post hoc* test: DLB > controls, AD (*p* < 0.001), controls versus AD (*p* = 0.14) (Mann–Whitney *U*).

### Voxel grey matter analysis

**Figure 2:**
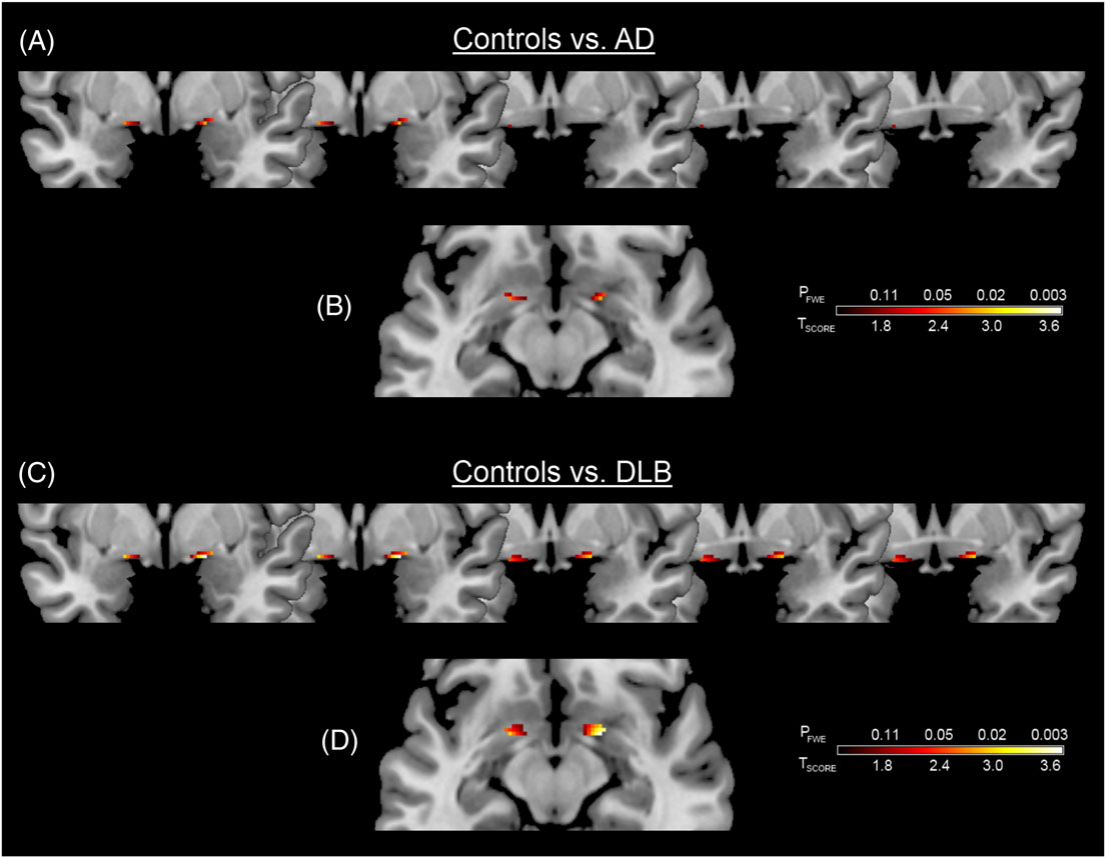
Significant grey matter loss of the substantia innominata in Alzheimer's disease (AD) and dementia with Lewy bodies (DLB) compared with controls. Results are superimposed on a magnetic resonance imaging T1 brain template image and displayed neurologically (left is left) in the coronal (A, C) and axial (B, D) views. [Colour figure can be viewed at wileyonlinelibrary.com]

SPM8 analysis showed significant bilateral GM loss (*p*_FWE_ ≤ 0.05) in the SI in AD compared to controls (Figure 2(A, B) and Table 2). Bilateral GM loss in the SI was also apparent in DLB, compared to controls (*p*_FWE_ ≤ 0.05; Figure 2(C, D) and Table 2). No significant differences were observed between AD and DLB for either contrast (AD > DLB or DLB > AD), and no significant SI atrophy was found in controls that exceeded AD or DLB. Of note, results did not significantly differ when we controlled for the effect of the different MRI sequence used in the two groups of participants.

**Table 2:** Location and peak significance of voxel GM analyses in the substantia innominata using DARTEL‐VBM

	Voxel level (*p*_FWE‐corr_)	Extent (*k*)	*t*, *Z*	MNI coordinates (*x*, *y*, *z*) (mm)
Group effects				
Controls versus AD	0.03	9	2.8, 2.7	18, 2, −12
	0.04	8	2.6, 2.6	−18, 2, −12
Controls versus DLB	0.002	28	3.7, 3.6	18, 2, −12
	0.02	20	2.9, 2.9	−18, 2, −12
AD versus DLB			No significant differences	
Regression				
MMSE	0.05	9	2.7, 2.5	−9, 2, −12
CAF	0.05	36	−2.7, −2.5	14, 3, −12

GM, grey matter; DARTEL‐VBM, diffeomorphic anatomical registration through exponentiated lie algebra voxel‐based morphometry; MNI, Montreal Neurological Institute; AD, Alzheimer's disease; DLB, dementia with Lewy bodies; MMSE, mini‐mental state examination; CAF, Clinician Assessment of Fluctuations.

This table depicts the corrected voxel‐level significance (*p*_FWE‐corr_), spatial extent (*k*), *t* and *Z* scores and MNI coordinates.

### Regression analyses

**Figure 3:**
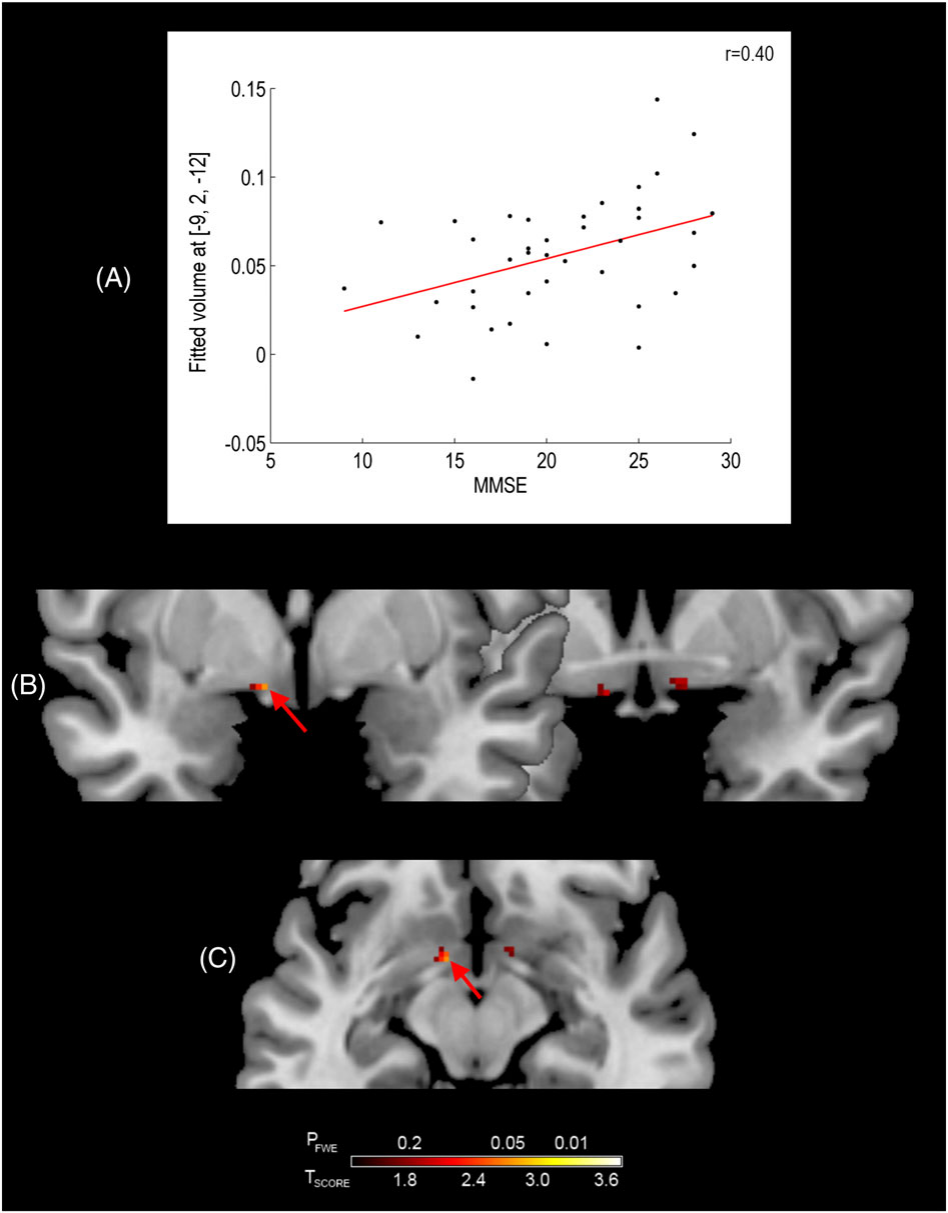
Association between substantia innominata grey matter volume and mini‐mental state examination (MMSE) in dementia with Lewy bodies. Graph shows the relationship at the most significant voxel (red arrow). Results are superimposed on a magnetic resonance imaging T1 brain template image and displayed neurologically (left is left) in the coronal (B) and axial (C) views. [Colour figure can be viewed at wileyonlinelibrary.com]

**Figure 4:**
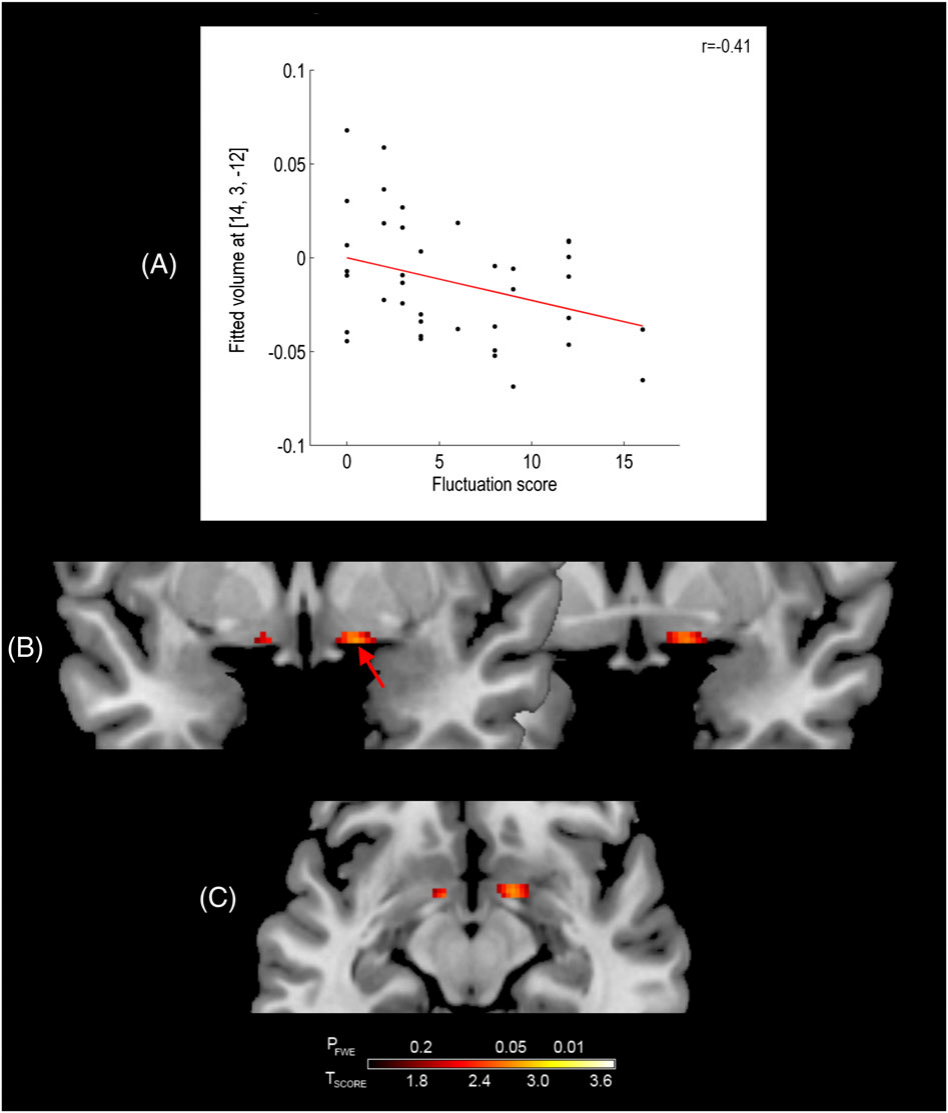
Association between substantia innominata grey matter volume and fluctuation score in dementia with Lewy bodies. The graph shows the relationship at the most significant voxel (red arrow). Results are superimposed on a magnetic resonance imaging T1 brain template image and displayed neurologically (left is left) in the coronal (B) and axial (C) views. [Colour figure can be viewed at wileyonlinelibrary.com]

The effects of SI volume loss upon MMSE, CAF, NPI and NPI hallucinations scores were separately evaluated in AD and DLB using multiple regression analyses, where age and TIV_SPM8_ were included as covariates. In DLB, a significant positive association (*p*_FWE_ ≤ 0.05) was shown between MMSE and GM volume in the left SI (Figure 3(B, C) and Table 2). In addition, a significant negative association (*p*_FWE_ ≤ 0.05) was also shown between CAF scores and GM volume in the right SI (Figure 4(B, C) and Table 2) in DLB. Associations between SI volume and total NPI scores, and between GM SI volume and NPI hallucination scores, were not statistically significant. There were no significant associations between any of these measures and GM SI volume in AD.

## Discussion

The present study examined GM volume changes exclusively within the SI in AD, DLB and similarly‐aged healthy individuals using DARTEL‐VBM. Three main findings emerge from this study. Firstly, GM loss was observed in both AD and DLB relative to healthy controls. Secondly, a positive association was observed between MMSE and SI volume in DLB, whilst lastly in the same group of patients, a negative association was shown between SI volume and CAF scores.

In the present study, SI volume was reduced in dementia groups compared with healthy controls, which was consistent with previous studies (Grothe *et al*., 2014; Hanyu *et al*., 2005; Whitwell *et al*., 2007). In addition, atrophic changes within the SI appeared to be associated with dementia severity and cognitive fluctuations in DLB. Deficits in cognition and cognitive fluctuations are key symptoms in DLB (McKeith *et al*., 2005), and whilst the cholinergic system has a key role in cognitive and attentional function (Baxter and Chiba, 1999; Niewiadomska *et al*., 2009), the influence of the cholinergic system upon cognitive fluctuations is not currently well understood in DLB. The present study therefore suggests that the SI contributes to the symptom of cognitive fluctuations in DLB, although other evidence has suggested that cognitive fluctuations may also depend upon changes in other brain networks and neurotransmitter systems, as well as cortico‐thalamic disturbances (Delli Pizzi *et al*., 2015; Francis *et al*., 2006; Peraza *et al*., 2014). There was no association between visual hallucinations and SI volume, despite the support for the role of cholinergic dysfunction in visual hallucinations (McKeith *et al*., 2000; Perry *et al*., 1990a). However, other cholinergic nuclei aside from the NBM may play a more prominent role in visual hallucinations; certainly, a previous VBM study showed that in PD patients with dementia, visual hallucinations were associated with pedunculopontine nucleus atrophy (Janzen *et al*., 2012).

Strengths of the current study include the relatively large AD and DLB cohorts, the acquisition of higher‐field 3 T MRI data compared with previous studies assessing the SI in DLB (Hanyu *et al*., 2005; Whitwell *et al*., 2007), the use of rigorous and validated methodologies for imaging and the extensive clinical and cognitive profiling of participants. There are several study limitations. Firstly, despite following a previously reported protocol (Choi *et al*., 2012; George *et al*., 2011; Shin *et al*., 2012), these findings should still be considered tentative, given the relatively small size, proximity and subsequent methodological challenges surrounding the automated GM segmentation of the SI and of similar structures from MR images. Secondly, there were no pathologically confirmed diagnoses, although the applied clinical diagnostic criteria were associated with high diagnostic specificity (Ferman *et al*., 2011; Knopman *et al*., 2001). Lastly, although the examination of a significant omnibus effect across groups, followed by appropriate *post hoc* tests, would have allowed a more systematic approach to the group analyses, this approach is potentially too conservative, and where focal changes between different types of dementia and healthy ageing are often relatively small, potentially important findings can be overlooked. Whilst not statistically significant, the GM loss appeared to be more widespread in DLB than in AD, and this may reflect the heterogeneity of the DLB patient group.

In summary, the present study examined the GM volume changes in the SI in AD, DLB and similarly aged healthy individuals using DARTEL‐VBM. These results suggest that GM atrophy and the clinical correlates of the SI may be important in understanding some of the clinical manifestations of DLB, which warrants further investigation.

## Conflict of interest

None declared.

**Key points**

- It is unclear to what extent cognitive deficits and cognitive fluctuations in dementia with Lewy bodies depend upon the nucleus basalis of Meynert.
- The extent of substantia innominata grey matter loss, relative to controls, was greater in dementia with Lewy bodies than Alzheimer's disease.
- Significant associations between substantia innominata grey matter volume loss and clinical measures were observed.
